# Changing Trends in Melanoma Incidence and Decreasing Melanoma Mortality in Hungary Between 2011 and 2019: A Nationwide Epidemiological Study

**DOI:** 10.3389/fonc.2020.612459

**Published:** 2021-02-12

**Authors:** Gabriella Liszkay, Zoltan Kiss, Roland Gyulai, Judit Oláh, Péter Holló, Gabriella Emri, András Csejtei, István Kenessey, Angela Benedek, Zoltán Polányi, Zsófia Nagy-Erdei, Andrea Daniel, Kata Knollmajer, Máté Várnai, Zoltán Vokó, Balázs Nagy, György Rokszin, Ibolya Fábián, Zsófia Barcza, Csaba Polgár

**Affiliations:** ^1^Department of Dermato-Oncology, National Institute of Oncology, Budapest, Hungary; ^2^MSD Pharma Hungary Ltd., Budapest, Hungary; ^3^Department of Dermatology, Venereology and Oncodermatology, Faculty of Medicine, University of Pécs, Pécs, Hungary; ^4^Department of Oncotherapy, University of Szeged, Szeged, Hungary; ^5^Department of Dermatology and Allergology, University of Szeged, Szeged, Hungary; ^6^Department of Dermatology, Venereology and Dermatooncology, Semmelweis University, Budapest, Hungary; ^7^Department of Dermatology, University of Debrecen, Debrecen, Hungary; ^8^Department of Oncoradiology, Markusovszky University Teaching Hospital, Szombathely, Hungary; ^9^Center for Health Technology Assessment, Semmelweis University, Budapest, Hungary; ^10^RxTarget Ltd., Szolnok, Hungary; ^11^Biomathematics and Informatics Department, University of Veterinary Medicine, Budapest, Hungary; ^12^Syntesia Medical Communications Ltd., Budapest, Hungary; ^13^Centre of Radiotherapy, National Institute of Oncology, Budapest, Hungary; ^14^Department of Oncology, Faculty of Medicine, Semmelweis University, Budapest, Hungary

**Keywords:** melanoma, incidence, mortality, trend change, Hungary

## Abstract

**Background:**

The incidence of malignant melanoma has continually increased during the past few decades, however, certain reports suggest a recent change in trends. The aim of our study was to examine the epidemiology of melanoma in Hungary.

**Methods:**

This nationwide, retrospective, longitudinal study included melanoma patients diagnosed between 1 January 2009 and 31 December 2019 using the databases of the National Health Insurance Fund (NHIF) and Central Statistical Office (CSO) of Hungary. Age-standardized incidence and cause-specific mortality rates were calculated.

**Results:**

We identified 2,426 and 2,414 new melanoma cases in 2011 and in 2019. Age-standardized incidence rates were higher in males and varied between 28.28 and 34.57/100,000 person-years (PYs), and between 22.63 and 26.72/100,000 PYs in females. We found 16.14 and 18.82% increases in male and female incidence rates from 2011 to 2015 (p=0.067 and p<0.001, respectively), and 12.77 and 11.35% decreases from 2015 to 2019 (p=0.062 and p=0.004, respectively). The change of incidence trends (2011–2015 vs. 2015–2019) was significant in females (p=0.002) and in the total melanoma population (p=0.011), but not in the male population (p=0.063). A 16.55% (95% CI: −27.07 to −4.59; p=0.013) decrease in mortality rates was found in the overall melanoma population.

**Conclusions:**

We observed a significant trend change in melanoma incidence in the female and total melanoma population, and a significant decrease in mortality in the total melanoma population. These changes may be attributed to intensive melanoma awareness campaigns as well as to the increase in screening and access to modern therapies.

## Introduction

The incidence of malignant melanoma has shown a continuous increase worldwide among light-skinned populations during the past few decades (1–5). In Europe, the estimated age-standardized rates (ASRs per 100,000 person-years, PYs) of melanoma incidence increased from 11.4 in males and 11.0 in females in 2012 to 15.8 and 14.6 in 2018, respectively (6, 7). Although incidence rates are still increasing in most European countries, certain high-risk countries, including Australia, Scandinavia, the United Kingdom and the United States have reported a levelling off or stabilization in melanoma incidence rates mainly in younger age groups starting from the late 1990s in contrast to the dramatic increase during the preceding years (1, 3, 8–11). Due to the significant burden of the disease, several countries have implemented educational programs and prevention campaigns, the long-term impact of which are yet to be seen during the upcoming years (12, 13).

Parallel to the overall increasing incidence, the mortality of melanoma has also been reported to increase in the past few decades (4, 5, 14, 15). However, the increase in mortality also seems to have decelerated or halted in certain regions, and several recent reports even suggest a slight decrease in younger age groups, which may be attributed to the introduction of modern therapies as well as to screening, improved early recognition and the implementation of awareness programs (9, 16–20).

Based on the estimations by Ferlay et al., Hungary was in the middle rank of the spectrum of melanoma incidence and mortality both in 2012 and 2018 (6, 7). It should be noted that the incidence rates for Hungary were estimated based on mortality-to-incidence (M/I) ratios from neighboring countries and thus cannot be considered an accurate and appropriate basis for evaluation.

Therefore, our current population-based analysis aimed to examine the incidence and mortality of melanoma in Hungary between 2011 and 2019, and to compare data with international findings from the same period.

## Materials and Methods

### Study Design

Our nationwide, retrospective, longitudinal study was conducted using the databases of the National Health Insurance Fund of Hungary (NHIF) and Central Statistical Office (CSO). The NHIF database is a comprehensive database covering close to 100% of the Hungarian population which contains prescription claims data from all reimbursed medicinal products as well as ICD-10 code information about all in- and out-patient visits. The CSO database collects cause-specific mortality data on all deceased Hungarian citizens on a yearly basis.

The present analysis included patients with malignant melanoma (ICD-10 C43) who were diagnosed between 1 January 2009 and 31 December 2019 and were ≥20 years of age at the time of diagnosis. To avoid the potential miscoding of melanoma, we included patients with a minimum of two occurrences of the ICD-10 code C43. Only one occurrence of C43 was also accepted if the patient died within 60 days after the first ICD-10 code. The 2-year-long period between 2009 and 2010 was considered a reference period to detect newly diagnosed melanoma patients from 2011. Hungarian population sizes for incidence and prevalence calculations by age and sex, as well as dates and numbers of cause-specific mortality among melanoma patients were obtained from the Hungarian CSO. All-cause mortality data were retrieved from the NHIF database for the cumulative prevalent melanoma population. Thus, we are able to calculate all-cause mortality as well as cause-specific mortality data for the melanoma population on a yearly basis.

For the calculation of incidence rates, annual numbers of patients newly diagnosed with melanoma are given as crude numbers (n), new cases were counted for each calendar year, (between 1 January and 31 December). Annual incidence rates are expressed as standardized rates (per 100,000 PYs). In addition, we also calculated annual cumulative incidence as percentages (%) of the total population at risk. Total population at risk was determined by subtracting the number of prevalent melanoma cases known on 1 January of a given year from the total population of the same year based on annual mid-year population estimates from the CSO.

For prevalence calculations, the number of melanoma patients was determined using the annual number of melanoma patients who were alive on 1 January of the given year. Patients newly diagnosed in the given year were also included in the annual prevalence. Annual prevalence was expressed as crude numbers (n), in addition, we also calculated prevalence rates as percentages (%) of the total population based on annual mid-year population estimates from the CSO (21). Age-standardized prevalence per 100,000 PYs were also calculated by sex using the cohort weights from European Standard Population (ESP) 2013.

The calculation of cause-specific mortality rates was based on data from the CSO database. We considered the number of patients who died of melanoma between 1 January and 31 December of a given year as the number of melanoma cause-specific deaths. Melanoma-specific mortality was expressed as crude numbers (n) and standardized rates per 100,000 PYs. We used standardized incidence and cause-specific mortality rates to evaluate trends in incidence and mortality over time. Total changes and annual changes between 2011–2019, 2011–2015 and 2015–2019 were presented as percentages (%).

To allow for direct comparisons with recent and earlier publications, incidence and mortality data were adjusted for age using both ESP 1976 and 2013 for standardization. Where crude numbers of any parameter were recorded below 10, we indicated “<10” as the NHIF data protection rule does not allow the presentation of case numbers below 10 in a stratum. In these cases, calculations were run on the exact crude numbers. The study protocol was approved by the National Ethical Board for Health Research (IV2581-2/2020/EKU).

### Statistical Analysis

Regression models were used to estimate annual trends with 95% confidence intervals (95% CI). As data were not independent, a block-based bootstrap method was used for time series with a fixed block size of 2. Hungarian population sizes were calculated based on mid-year population sizes published by the Hungarian CSO. The size of the at-risk population was determined based on the difference between mid-year population sizes and the number of previously diagnosed melanoma patients on 1 January in a given year. Poisson regression was used for the calculations of annual change of incidence and mortality. In the 2011 to 2019 period the outcome was the number of patients, the offset was the log of the number of patients at risk or the mid-year population, the explanatory variables were the year. When comparing the periods 2011–2015 and 2015–2019, the outcome was the number of patients, the offset was the log of the number of patients at risk or the mid-year population, the two explanatory variables were the number of years since 2011 and the number of years since 2015. All calculations were performed with R version 3.6.1 (05/07/2019) with package boot version 1.3-20.

## Results

### Crude Numbers

**Figure 1:**
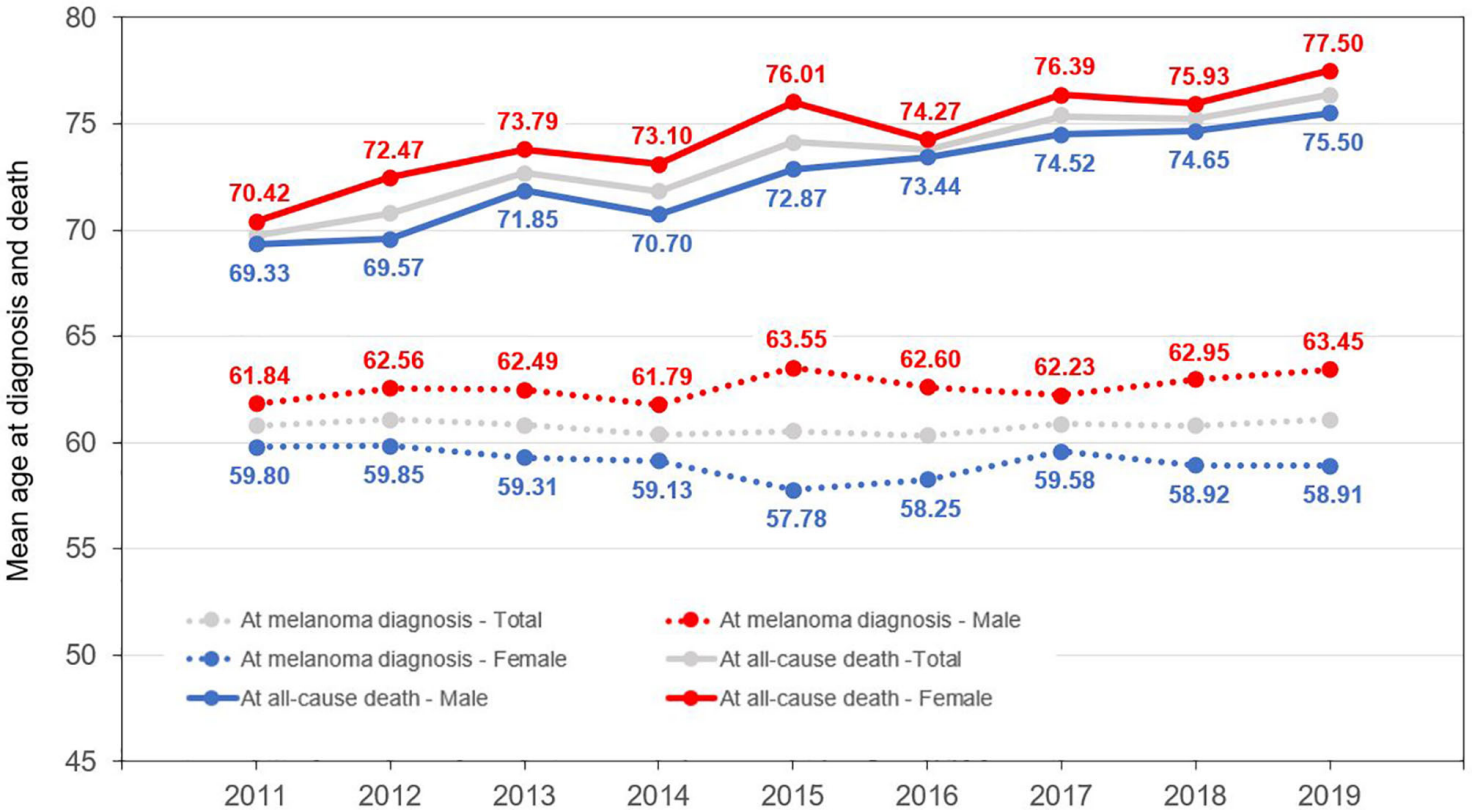
Mean age of melanoma patients at diagnosis and at the time of death (all-cause mortality).

We identified 2,426 and 2,414 new melanoma cases in 2011 and 2019 from the NHIF database, respectively, corresponding to 0.02–0.03% of the total Hungarian population at risk (**Table 1**) The proportion of male patients varied from 49.51 to 45.64% between 2011 and 2019. The mean age at diagnosis was 61.84 years in men (SD ± 15.18) and 59.80 years in women (SD ± 16.80) in 2011, and 63.45 (SD ± 15.08) and 58.91 years (SD ± 16.17) in 2019 (**Figure 1**, **Supplementary Table 1**). The total number of identified melanoma patients increased from 15,388 to 28,660 during the study period, with female dominance (56.99 to 57.54%) (**Table 1**).

**Table 1:** Number of incident and prevalent melanoma cases by sex, and melanoma cause-specific and all-cause mortality of melanoma patients (melanoma cause-specific mortality for 2019 is not available in the CSO database yet).

Incident population	2011		2012		2013		2014		2015		2016		2017		2018		2019	
Patients with new Melanoma diagnosis (n, % of population at risk)	2,426	0.02%	2,360	0.02%	2,503	0.03%	2,557	0.03%	2,803	0.03%	2,684	0.03%	2,673	0.03%	2,528	0.03%	2,414	0.02%
Male (n, % of new M patients)	1,201	49.51%	1,077	45.64%	1,201	47.98%	1,221	47.75%	1,346	48.02%	1,279	47.65%	1,314	49.16%	1,182	46.76%	1,169	48.43%
Female (n, % of new M patients)	1,225	50.49%	1,283	54.36%	1,302	52.02%	1,336	52.25%	1,457	51.98%	1,405	52.35%	1,359	50.84%	1,346	53.24%	1,245	51.57%
**Prevalent population**																		
Patients with Melanoma diagnosis (n, % of total population)	15,388	0.15%	17,041	0.17%	18,773	0.19%	20,546	0.21%	22,481	0.23%	24,228	0.25%	25,939	0.27%	27,365	0.28%	28,660	0.29%
Male (n, % of new M patients)	6,582	42.77%	7,235	42.46%	7,994	42.58%	8,779	42.73%	9,662	42.98%	10,387	42.87%	11,156	43.01%	11,728	42.86%	12,277	42.84%
Female (n, % of new M patients)	8,806	57.23%	9,806	57.54%	10,779	57.42%	11,767	57.27%	12,819	57.02%	13,841	57.13%	14,783	56.99%	15,637	57.14%	16,383	57.16%
**Cause specific mortality**																		
Patients died based on CSO (n, % of prevalent population)	363	2.36%	377	2.21%	347	1.85%	378	1.84%	349	1.55%	337	1.39%	333	1.28%	317	1.16%		
Male (n, % of new M patients)	213	58.68%	210	55.70%	184	53.03%	193	51.06%	215	61.60%	198	58.75%	188	56.46%	177	55.84%		
Female (n, % of new M patients)	150	41.32%	167	44.30%	163	46.97%	185	48.94%	134	38.40%	139	41.25%	145	43.54%	140	44.16%		
**All-cause mortality**	**2011**		**2012**		**2013**		**2014**		**2015**		**2016**		**2017**		**2018**		**2019**	
Patients died based on NHIF (n, % of prevalent population)	707	4.59%	771	4.52%	784	4.18%	868	4.22%	937	4.17%	962	3.97%	1,102	4.25%	1,119	4.09%	1,183	4.13%
Male (n, % of new M patients)	424	59.97%	442	57.33%	436	55.61%	463	53.34%	554	59.12%	545	56.65%	610	55.35%	620	55.41%	665	56.21%
Female (n, % of new M patients)	283	40.03%	329	42.67%	348	44.39%	405	46.66%	383	40.88%	417	43.35%	492	44.65%	499	44.59%	518	43.79%

CSO, Central Statistical Office; M, melanoma; NHIF, National Health Insurance Fund.

The annual number of melanoma patients who died from any cause increased from 707 (2011) to 1,183 (2019), while melanoma cause-specific mortality decreased from 365 to 318 per year during the study period. The mean age at the time of all-cause death increased from 69.77 (SD ± 13.14) to 76.37 (SD ± 12.26) years between 2011 and 2019, and was higher among females during the whole study period (70.42–77.50 years vs. 69.33–74.65 years in males) (**Figure 1**, **Supplementary Table 1**)

### Incidence

**Figure 2:**
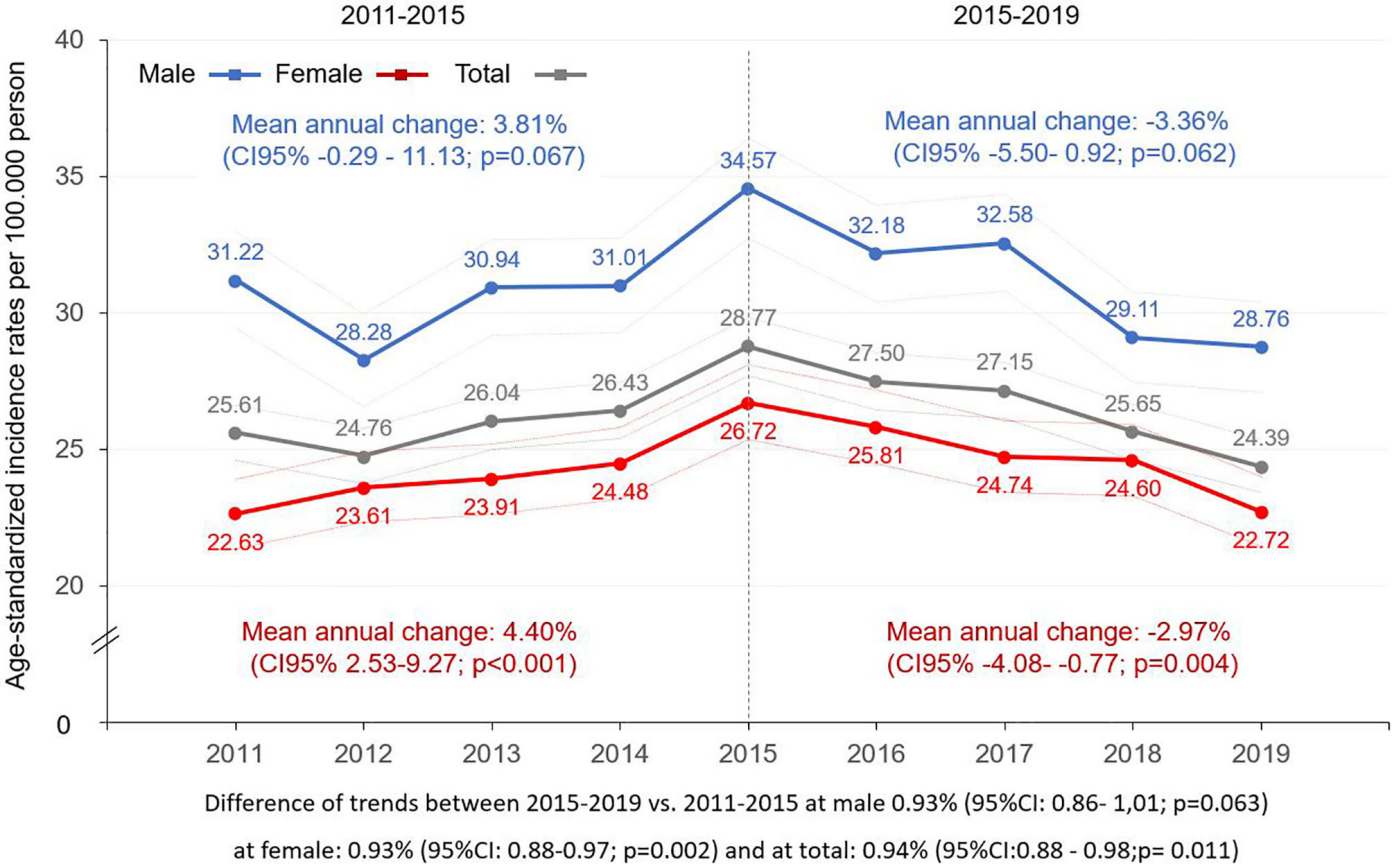
Age-standardized incidence rates (ESP 2013) of melanoma (C43) by sex in Hungary between 2011 and 2019 (per 100,000 person-years; dotted lines represent 95% CI). CI, confidence interval; ESP, European Standard Population.

Age-standardized incidence rates (ESP 2013) were higher in males during the whole study period, with the lowest rate found in 2012 (28.28/100,000 PYs; 95% CI: 26.59–29.97) and highest in 2015 (34.57/100,000 PYs, 95% CI: 32.73–36.42) (**Figure 2**, **Supplementary Table 2**). We found a 16.14% increase in incidence rates between 2011 and 2015 in males (mean annual change: 3.81 (95% CI: −0.29–11.13); p=0.067) (**Supplementary Table 3**). Conversely, a 12.77% decrease was seen during the 2015–2019 period (mean annual change: −3.36%; 95% CI: −5.50–0.92; p=0.062). Age-standardized incidence rates in females increased by 18.82% from 22.63 to 26.72/100,000 PYs in the 2011–2015 period (95% CI: 10.5–42.58; p<0.001), then decreased by 11.35% to 22.72/100,000 PYs (95% CI: −15.35 to −3.05; p=0.004) by 2019. Adjusted incidence rates for the total melanoma population increased by 3.76% yearly (95% CI: 1.28–8.87; p=0.009) in the first, and decreased by 3.00% (95% CI: −4.18 to −0.63; p=0.035) in the second period (**Supplementary Table 3**). The analysis of trends in the two study periods (2015–2019 vs. 2011–2015) revealed significant differences in females as well as in the total melanoma population (change in females: −7.06%; 95% CI: −11.77 to −3.54; p=0.002; change in total population: −6.51%; 95% CI: −11.65 to −2.23; p= 0.011), and a non-significant trend change in the male melanoma population (change: −6.91%; 95% CI: −14.23–0.69; p=0.063).

### Mortality

**Figure 3:**
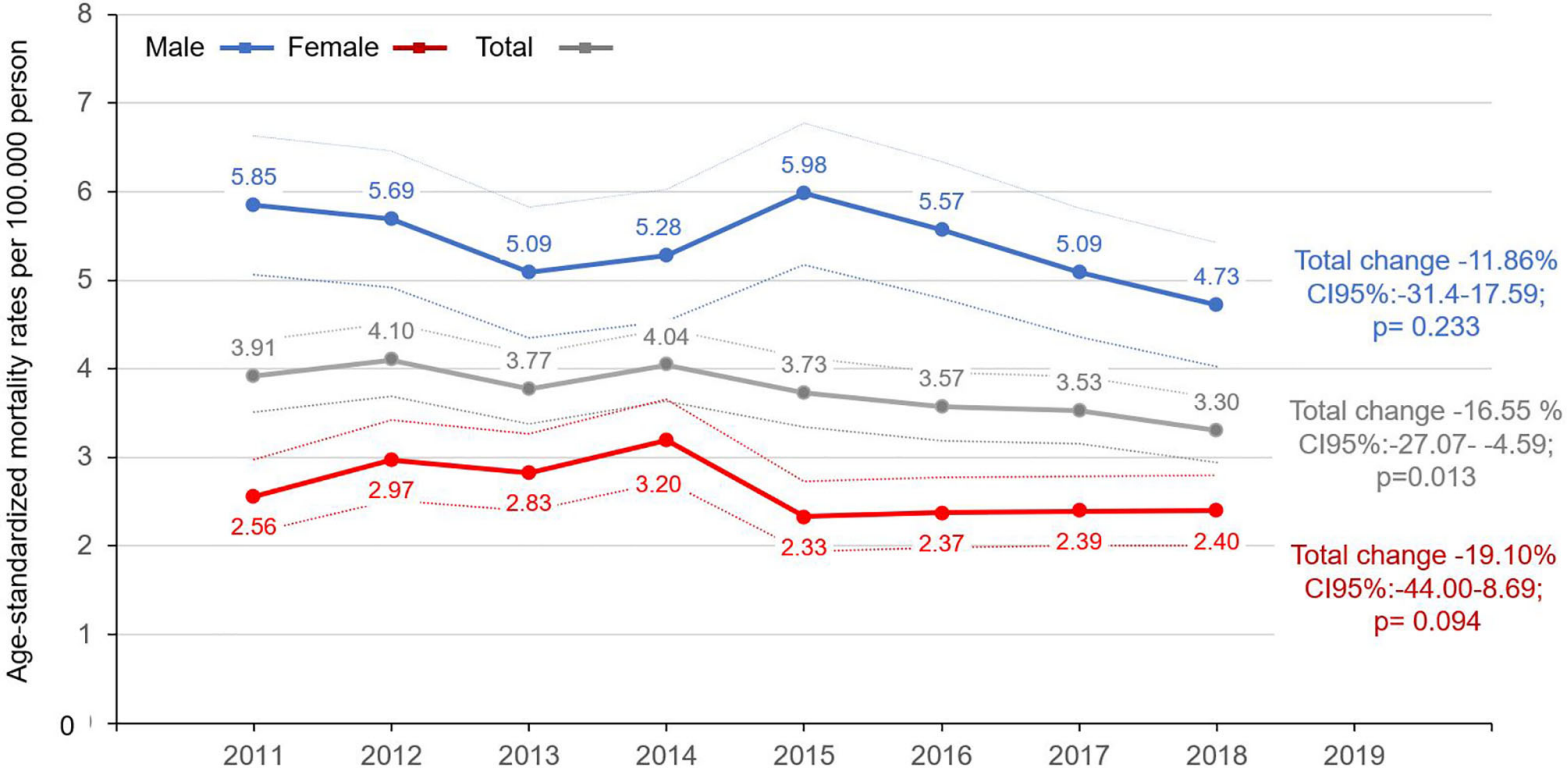
Age-standardized mortality rates (standard: ESP 2013) of melanoma patients by sex in Hungary between 2011 and 2018 (per 100,000 person-years; dotted lines represent the 95% CI). CI, confidence interval; ESP, European Standard Population.

Age-standardized, cause-specific mortality rates (ESP 2013) of melanoma varied between 5.98 (95% CI: 5.18–6.78) and 4.73 (95% CI: 4.03–5.42) per 100,000 PYs in men, and between 2.33 (95% CI: 1.94–2.73) and 3.20 (95% CI: 2.73–3.66) in women in the 2011–2018 period (**Figure 3**, **Supplementary Table 4**). There was no significant change in age-standardized cause-specific mortality rates in male patients from 2011 to 2018 (change: −11.86%; 95% CI: 31.4–17.59; p=0.233) or in the female melanoma population (change: −19.1%; 95% CI: −44.00–8.69; p=0.094). However, a significant decrease by 16.55% (95% CI: −27.07 to −4.59; p=0.013) was found during the 2011–2018 period in the overall melanoma population (**Supplementary Table 5**). We did not find any significant change in trends between the 2011–2015 and 2015–2018 periods in any of the sexes, nor in the total melanoma population (p=0.312, p=0.163, p=0.779 for the female, male, and overall populations, respectively) (**Supplementary Table 5**).

### Incidence and Mortality Compared to Other European Countries

**Figure 4:**
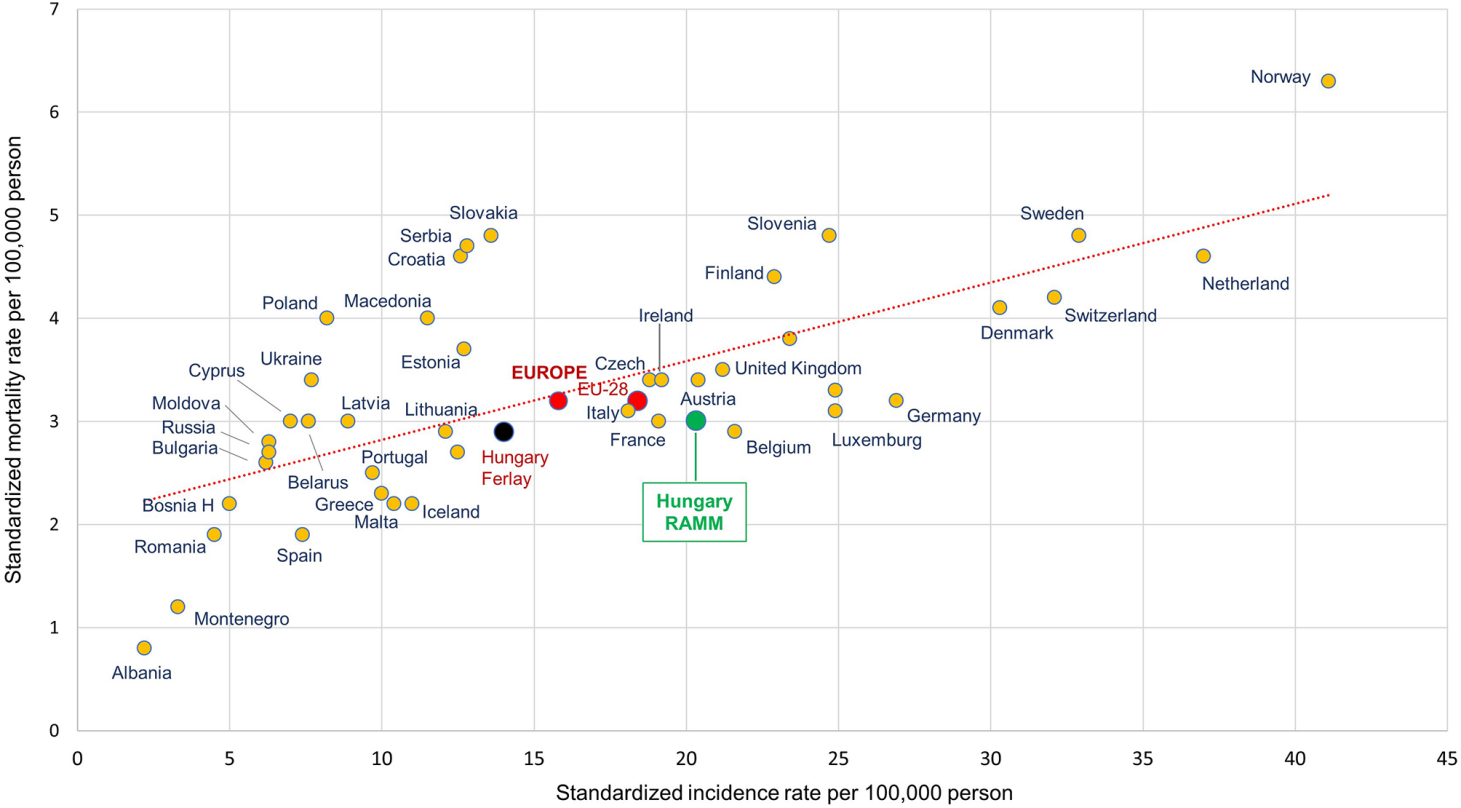
Age-standardized incidence and mortality rates per 100,000 person-years among male melanoma (C43) patients in European countries and Hungary based on the NHIF survey in 2018 (ESP 1976). ESP, European Standard Population. Description, A dot in the graph shows age-standardized incidence and mortality rates together for a country. The Y axis represents the standardized mortality rate per 100,000 population, while the X axis represents the standardized incidence rate per 100,000 population. (EU-28, 28 European member states; RAMM, name of current Hungarian study; Hungary Ferlay, reference numbers of Hungary from Ferlay publication).

In our current study, the age-standardized incidence of melanoma per 100,000 PYs was 20.41 in men (95% CI: 19.25–21.58), while age-standardized mortality was 3.01 (95% CI: 2.57–3.45) in 2018 (using ESP 1976 for standardization). These results are different from the data reported by Ferlay et al. for Hungary from the same year, with estimated ASRs of 14.0 for incidence and 2.9 for mortality (**Figure 4**). There was also a considerable difference between our results based on the NHIF database and the publication by Ferlay et al. among women: we found an incidence rate of 20.13 (95% CI: 19.06–21.21) and a mortality rate of 1.66 (95% CI: 1.38–1.93) in 2018 (**Supplementary Table 6**), while those reported by Ferlay et al. were 13.20 and 2.10, respectively. We also compared the age standardized incidence rates of European countries On **Supplementary Figure 1** for both sexes using the Ferlay reports from 2012 and 2018 (6, 7).

## Discussion

The main findings of our nationwide, long-term retrospective study can be summarized as follows:

1. We found strong statistical evidence for a change in the trend of age-standardized melanoma incidence starting from 2015.
2. Age-standardized cause-specific melanoma mortality rates decreased from 2011 to 2018 in the male, female, and overall melanoma populations, with a statistically significant decrease of 16.55% observed in the total population. The mean age of melanoma patients at the time of death increased by approximately 6–7 years during the study period in both sexes.

### Incidence

Melanoma has been reported as one of the most common cancer types among light-skinned people in recent years (7, 22–24). Several countries have reported a continuous increase in melanoma incidence during recent decades until the mid-2010s (25–27). The reasons for this increasing trend are multifactorial. Greater public awareness and increased detection as well as the development and broader use of high-accuracy diagnostic tools may explain some of the increase in incidence rates (25, 28, 29). However, a recent U.S. study reported increasing rates even in populations with limited access to quality healthcare, suggesting that at least some of the rise is due to a real increase in incidence (26). Increased exposure to UV radiation may be responsible for the largest proportion of the increase in incidence, which is in line with the fact that the most affected regions are circumpolar areas (30–32). Sun-seeking behaviour and the increasing use of tanning beds also contribute to the increasing incidence of melanoma, especially in younger age groups (33, 34). Furthermore, as melanoma incidence increases with age and the highest rates can be found among people over 80 years, an ageing population is also an important driver for the increase (11).

Our study reports age-standardized incidence rates from the very recent years. Since all melanoma cases registered in 2019 were already available in the NHIF database at the time of our manuscript submission, this study is among the very few reporting data on melanoma incidence from the second half of the 2010s. Our findings based on the NHIF database are supported by data from the Hungarian National Cancer Registry, despite differences in data collection methods: the difference in the annual number of newly diagnosed melanoma patients in the two data sources was below 5% (Hungarian National Cancer Registry) (35).

We found significant changes in melanoma incidence trends starting from 2015. Between 2011 and 2015, the mean annual increase in incidence was significant, 3.76% in the overall melanoma population. In contrast, relevant and statistically significant changes in trends were observed between 2015 and 2019, with a mean annual decrease of 3.00%. The change between the 2015–2019 and 2011–2015 periods was also significant.

Primary prevention has been in the focus of the Hungarian Society of Dermatology for decades. Hungary joined the Euromelanoma skin cancer prevention and education alliance formally in 2009 and enter the international scientific collaborations (36–38). Before this time, the Hungarian Society of Dermatology successfully organized and offered skin cancer screenings countrywide with public awareness campaign with the aims to stress the importance of sun protection and early recognition of suspicious malignant lesions. By joining to the Euromelanoma campaign, the Hungarian screening campaign reached even wider population, by 2013 15.000 people asked dermatological examination (39). Local implementation of the Euromelanoma program is based on local observations to increase its penetration into the lay community. As such, the health consciousness of the subgroup of people seeking dermatological screening under the umbrella of Euromelanoma at the University of Szeged was evaluated by a detailed questionnaire. They found that these patients were health-conscious regarding cardiovascular diseases and attend cancer screenings on recall. However, their knowledge about melanoma is insufficient, eg. most of them perform skin self-examination, but they did not know what to check, lesion was detected mostly by the patients themselves, but it took more than one year to consult a doctor. Our study confirmed that patients’ attitude toward melanoma is an important factor influencing early detection therefore which should be introduced into the routine medical care (40). In parallel with the above mentioned efforts made by the dermatologist community in Hungary, scientific reports evaluated the epidemiology of melanoma which showed gradual transformation of the body of incident melanoma cases over time. The National Institute of Oncology analysed cutaneous malignant melanoma cases over 10 years, between 1998 and 2008. Although the number of cutaneous melanomas showed an 153% increased between 1998 and in 2008, the mean of Breslow numbers decreased significantly from 2.2 mm to 1.6 mm (p = 0.0002). Clark numbers were also decreasing trend, although the difference was not significant (p = 0.08) (41). Another report investigated the newly recognized cutaneous melanoma in the University of Debrecen hospital-based registry in North-East Hungary from 2000 to 2014. A total of 1509 cutaneous invasive melanomas of 1464 patients were included in the study which showed a moderate but significant increase in incidence [average annual percentage change: 3.04 (0.07; 6.11); P = 0.045], with a breakpoint in 2007. From 2001 to 2007, the trend was increasing [APC: 9.84 (3.52; 16.55); P=0.006], but it stalled from 2007 [APC: −2.45 (−5.99; 1.23); P = 0.164]. They found that the increase in the incidence of cutaneous melanoma in the region from 2000 to 2008 was because of an increase in the number of diagnosed thin tumors (42). Based on the Hungarian National Meteorology Service database, the change in annual amounts of biologically effective UV radiation is significantly increasing from 1995 to 2010 period, where is a 2 years peak in 2010-2011 followed by a relevant and significant decrease (43).

The incidence rates found in our study are comparable to data from the Cancer Research UK database, with the same peaks observed in 2015 in men and 2016 in women, respectively (11). Although the majority of recent studies still report an increase in melanoma incidence (4, 5), the changes in incidence trends observed in the current analysis are not unprecedented. A comprehensive analysis by Erdmann et al. examined global patterns and trends in melanoma incidence in 39 countries between 1953 and 2008, and found a stabilization of incidence rates in the early 2000s in the white populations of Australia, New Zealand, and North America, Israel, Iceland, and Norway, with signs of a declining trend mainly in younger generations (25–44 years) (1). Accordingly, a U.S. analysis found statistically significant decreases in melanoma incidence for adolescents and young adults between 2006 and 2015 (44). Furthermore, although Ferlay’s articles on cancer incidence did not discuss changes in incidence between 2012 and 2018, they reported lower male and/or female melanoma incidence rates in 2018 compared to 2012 for certain European countries such as Latvia, Ireland, Croatia, Spain and Switzerland, using similar standardization. By comparing the 2018 and 2012 Ferlay reports on estimated melanoma incidence (**Supplementary Figure 1**), we could find increase at most countries, however there are some ones showing decrease during this period, like Slovakia, where incidence rate decreased from 14.9 onto 13.6 per 100.000 person at males and 12.1 onto 10.5 at females. We could demonstrate decrease in incidence in Iceland and Montenegro at both sex, in Croatia. There were no relevant changes in Czech, Slovakia, Spain, Portugal, Poland, Bulgaria, Russia, Romania and Albania, where the difference between two study years are less relevant than in other countries where the incidence increased significantly, like Belgium, Netherland and Norway (6, 7).

The change in incidence trends may be explained by the spread of melanoma awareness campaigns which are aimed at drawing attention to the role of UV radiation as a modifiable environmental factor in the development of melanoma. Australia, one of the most affected countries, has conducted a number of successful educational and awareness campaigns starting from the 1980s including the Healthy Text study and the SunSmart skin cancer prevention program (13, 45, 46). The Euromelanoma Skin Cancer Prevention Campaign founded in 1999 is now active in 33 European countries with public awareness campaigns and melanoma screening days, scientific publications and special events (12, 47–49). A decreasing trend in the overall use of tanning beds has been reported by a number of other studies recently, which may also be the result of awareness campaigns (50, 51). The estimations by Guy et al. suggest that a comprehensive skin cancer prevention program could help achieve a plateau in incidence and prevent 20% of melanoma cases between 2020 and 2030 among white males and females in the U.S. (3). Based on these publications and results from our study, a change in melanoma incidence can be expected in more and more countries in the upcoming decades.

### Mortality

Age-standardized melanoma mortality rates in the male and female populations were similar to the European average (6, 7). In addition, our study found a 16.55% (p=0.013) decrease in mortality in the total melanoma population from 2011 to 2018, although the change was not significant in males and females separately.

Decreasing trends in melanoma mortality have been reported by a number of studies, which can be attributed to earlier detection and the development of diagnostic modalities as well as the availability of life-prolonging modern therapies (18, 52). The real breakthrough in melanoma survival was brought about by the development of molecular targeted and immune checkpoint inhibitor therapies which have provided dramatic survival benefit for melanoma patients and are considered as standard-of-care treatment regimens for patients with metastatic melanoma (53, 54). As a result, improved survival has been demonstrated by an increasing number of real-world studies (17, 20, 55). However, certain countries still report increasing incidence and mortality rates, highlighting that there is still room for improvement in melanoma prevention and management (4, 5).

### Incidence and Mortality Rate of Hungary in Accordance of European Countries

We found higher incidence rate of melanoma in Hungary for 2018 as it was presented before in Ferlay publication (7). The base of main difference is due to the method how Ferlay estimated the incidence data for those countries, which did not report any incidence data for melanoma (and other cancer types). Hence, Ferlay used the mortality to incidence rate ratios of neighbourhood countries and applied to the reported mortality data of Hungary. Therefore, measured incidence of melanoma in Hungary could differ from previous estimations. On the other hand, we did not find relevant difference between Ferlay and our data in case of mortality rates, as we used the same source, the Hungarian Centre of Statistic Office. Nevertheless, the presentation of incidence and mortality rates on **Figure 4** demonstrate that melanoma burden in Hungary is close to Europe and EU 28 member states’ average as well as to Slovenia, Slovakia and Czech, which neighbourhood countries are on the same latitude than Hungary.

Our study has certain strengths and limitations. The nationwide nature of the NHIF database allowed for the inclusion of all melanoma patients diagnosed between 2011 and 2019 in Hungary and thus provides a robust basis for our study. However, the database does not contain any information on patient characteristics, laboratory data, vital signs, or baseline prognostic features.

## Conclusion

Our study is the first nationwide analysis describing the incidence and mortality of melanoma in Hungary during the very recent years. Incidence rates were slightly higher compared to previous reports, while mortality rates were similar. We observed significant trend changes in melanoma incidence and mortality starting from 2015, which may be attributed to intensive melanoma awareness campaigns as well as to the increase in screening and access to modern therapies.

## Data Availability Statement

The original contributions presented in the study are included in the article/**Supplementary Material**. Further inquiries can be directed to the corresponding author.

## Ethics Statement

The study protocol was approved by the National Ethical Board for Health Research (IV2581-2/2020/EKU).

## Author Contributions

GL, ZK: Conceptualization, methodology, writing—original draft. CP: Supervision, writing—review and editing. RG, JO, PH, GE, AC: Conceptualization, validation. IK: Data validation. AB, ZP, ZN-E, AD, MV: Conceptualization, validation of data. ZV, BN: Methodology, supervision. GR, IF: Data curation. ZB: Writing—review and editing. KK: Managing manuscript. All authors contributed to the article and approved the submitted version.

## Funding

The authors declare that this study received funding from MSD Pharma Hungary. The funder had the following involvement with the study: in study design, data collection and analysis, decision to publish, or preparation of the manuscript.

## Conflict of Interest

ZK, ZP, MV, AD, AB, KK, and ZN-E were employed by the company MSD Pharma Hungary. GR and IF were employed by the company RxTarget Ltd. ZB was employed by the company Syntesia Medical Communications Ltd.

The remaining authors declare that the research was conducted in the absence of any commercial or financial relationships that could be construed as a potential conflict of interest.

## Supplementary Material

Supplementary Figure 1 Melanoma incidence rate of European countries in 2012 and 2018 by sex based on Ferlay reports (6, 7).

## References

[1] Erdmann F Lortet-Tieulent J Schüz J Zeeb H Greinert R Breitbart EW. International Trends in the Incidence of Malignant Melanoma 1953-2008–are Recent Generations at Higher or Lower Risk? Int J Cancer (2013) 132(2):385–400. 10.1002/ijc.27616 22532371

[2] Arnold M Holterhues C Hollestein LM Coebergh JWW Nijsten T Pukkala E. Trends in incidence and predictions of cutaneous melanoma across Europe up to 2015. J Eur Acad Dermatol Venereol (2014) 28(9):1170–8. 10.1111/jdv.12236 23962170

[3] Guy GP Jr Thomas CC Thompson T Watson M Massetti GM Richardson LC. Vital Signs: Melanoma Incidence and Mortality Trends and Projections - United States, 1982-2030. MMWR Morb Mortal Wkly Rep (2015) 64(21):591–6. PMC4584771 26042651

[4] Sacchetto L Zanetti R Comber H Bouchardy C Brewster DH Broganelli P. Trends in Incidence of Thick, Thin and in Situ Melanoma in Europe. Eur J Cancer (2018) 92:108–18. 10.1016/j.ejca.2017.12.024 29395684

[5] Garbe C Keim U Eigentler TK Amaral T Katalinic A Holleczek B. Time Trends in Incidence and Mortality of Cutaneous Melanoma in Germany. J Eur Acad Dermatol Venereol (2019) 33(7):1272–80. 10.1111/jdv.15322 30387899

[6] Ferlay J Steliarova-Foucher E Lortet-Tieulent J Rosso S Coebergh JWW Comber H. Cancer Incidence and Mortality Patterns in Europe: Estimates for 40 Countries in 2012. Eur J Cancer (2013) 49(6):1374–403. 10.1016/j.ejca.2012.12.027 23485231

[7] Ferlay J Colombet M Soerjomataram I Dyba T Randi G Bettio M. Cancer Incidence and Mortality Patterns in Europe: Estimates for 40 Countries and 25 Major Cancers in 2018. Eur J Cancer (2018) 103:356–87. 10.1016/j.ejca.2018.07.005 30100160

[8] Hall HI Miller DR Rogers JD Bewerse B. Update on the Incidence and Mortality From Melanoma in the United States. J Am Acad Dermatol (1999) 40(1):35–42. 10.1016/S0190-9622(99)70562-1 9922010

[9] de Vries E Bray FI Coebergh JWW Parkin DM. Changing Epidemiology of Malignant Cutaneous Melanoma in Europe 1953-1997: Rising Trends in Incidence and Mortality but Recent Stabilizations in Western Europe and Decreases in Scandinavia. Int J Cancer (2003) 107(1):119–26. 10.1002/ijc.11360 12925966

[10] Australian Institute of Health and Welfare. Skin cancer in Australia. Cat. no. CAN 96 (2016). Canberra, Australia: AIHW. Available at: https://www.aihw.gov.au/getmedia/0368fb8b-10ef-4631-aa14-cb6d55043e4b/18197.pdf.aspx?inline=true (Accessed 29 May 2020).

[11] Cancer Research UK. Melanoma skin cancer incidence trends over time (2020). Available at: https://www.cancerresearchuk.org/health-professional/cancer-statistics/statistics-by-cancer-type/melanoma-skin-cancer/incidence#heading-Two (Accessed 26 May 2020).

[12] van der Leest RJT de Vries E Bulliard J-L Paoli J Peris K Stratigos AJ. The Euromelanoma Skin Cancer Prevention Campaign in Europe: Characteristics and Results of 2009 and 2010. J Eur Acad Dermatol Venereol (2011) 25(12):1455–65. 10.1111/j.1468-3083.2011.04228.x 21951235

[13] Tabbakh T Volkov A Wakefield M Dobbinson S. Implementation of the SunSmart Program and Population Sun Protection Behaviour in Melbourne, Australia: Results From Cross-Sectional Summer Surveys From 1987 to 2017. PloS Med (2019) 16(10):e1002932. 10.1371/journal.pmed.1002932 31593565 PMC6782093

[14] Whiteman DC Bray CA Siskind V Green AC Hole DJ Macke RM. Changes in the Incidence of Cutaneous Melanoma in the West of Scotland and Queensland, Australia: Hope for Health Promotion? Eur J Cancer Prev (2008) 17(3):243–50. 10.1097/CEJ.0b013e3282b6fe3f 18414196

[15] Sneyd MJ Cox B. A Comparison of Trends in Melanoma Mortality in New Zealand and Australia: The Two Countries With the Highest Melanoma Incidence and Mortality in the World. BMC Cancer (2013) 13:372. 10.1186/1471-2407-13-372 23915380 PMC3750694

[16] Garbe C Leiter U. Melanoma Epidemiology and Trends. Clin Dermatol (2009) 27(1):3–9. 10.1016/j.clindermatol.2008.09.001 19095149

[17] Dobry AS Zogg CK Hodi FS Smith TR Ott PA Iorgulescu JB. Management of Metastatic Melanoma: Improved Survival in a National Cohort Following the Approvals of Checkpoint Blockade Immunotherapies and Targeted Therapies. Cancer Immunol Immunother (2018) 67(12):1833–44. 10.1007/s00262-018-2241-x PMC6249064 30191256

[18] Gutiérrez-González E López-Abente G Aragonés N Pollán M Pastor-Barriuso R Sánchez MJ. Trends in Mortality From Cutaneous Malignant Melanoma in Spain (1982-2016): Sex-Specific Age-Cohort-Period Effects. J Eur Acad Dermatol Venereol (2019) 33(8):1522–8. 10.1111/jdv.15565 30868690

[19] Ward EM Sherman RL Henley SJ Jemal A Siegel DA Feuer EJ. Annual Report to the Nation on the Status of Cancer, Featuring Cancer in Men and Women Age 20-49 Years. J Natl Cancer Inst (2019) 111(12):1279–97. 10.1093/jnci/djz106 PMC6910179 31145458

[20] Berk-Krauss J Stein JA Weber J Polsky D Geller AC. New Systematic Therapies and Trends in Cutaneous Melanoma Deaths Among US Whites, 1986-2016. Am J Public Health (2020) 110(5):731–3. 10.2105/AJPH.2020.305567 PMC7144422 32191523

[21] CSO database source. http://statinfo.ksh.hu/Statinfo/themeSelector.jsp?lang=hu.

[22] Australian Institute of Health and Welfare. Cancer in Australia: An overview, 2014. AIHW cat.no. CAN 88. Canberra, Australia (2014). Available at: www.aihw.gov.au (Accessed 11 June 2020).

[23] Bray F Ferlay J Soerjomataram I Siegel RL Torre LA Jemal A. Global Cancer Statistics 2018: GLOBOCAN estimates of incidence and mortality worldwide for 36 cancers in 185 countries. CA Cancer J Clin (2018) 68(6):394–424. 10.3322/caac.21492 30207593

[24] Surveillance, Epidemiology, and End Results (SEER) Program. Cancer Facts: Melanoma of the skin. Available at: https://seer.cancer.gov/statfacts/html/melan.html (Accessed 11 June 2020).

[25] Bataille V de Vries E. Melanoma—Part 1: epidemiology, risk factors, and prevention. BMJ (2008) 337:a2249. 10.1136/bmj.a2249 19022841

[26] Linos E Swetter SM Cockburn MG Colditz GA Clarke CA. Increasing burden of melanoma in the United States. J Invest Dermatol (2009) 129:1666–74. 10.1038/jid.2008.423 PMC2866180 19131946

[27] Siegel RL Miller KD Jemal A. Cancer statistics, 2017. CA Cancer J Clin (2017) 67(1):7–30. 10.3322/caac.21387 28055103

[28] Seité S Del Marmol V Moyal D Friedman AJ. Public primary and secondary skin cancer prevention, perceptions and knowledge: an international cross-sectional survey. J Eur Acad Dermatol Venereol (2017) 31(5):815–20. 10.1111/jdv.14104 PMC6084324 28045207

[29] Marchetti MA Liopyris K Dusza SW Codella NCF Gutman DA Helba B. Computer algorithms show potential for improving dermatologists’ accuracy to diagnose cutaneous melanoma: Results of the International Skin Imaging Collaboration 2017. J Am Acad Dermatol (2020) 82(3):622–7. 10.1016/j.jaad.2019.07.016 PMC7006718 31306724

[30] Oikarinen A Raitio A. Melanoma and other skin cancers in circumpolar areas. Int J Circumpolar Health (2000) 59(1):52–6. 10850007

[31] Mettlin CJ. Skin cancer and ozone depletion: the case for global action. J Surg Oncol (2001) 77(2):76–8. 10.1002/jso.1072 11398156

[32] Leiter U Garbe C. Epidemiology of melanoma and nonmelanoma skin cancer–the role of sunlight. Adv Exp Med Biol (2008) 624:89–103. 10.1007/978-0-387-77574-6_8 18348450

[33] de Vries E Coebergh JW. Cutaneous malignant melanoma in Europe. Eur J Cancer (2004) 40:2355–66. 10.1016/j.ejca.2004.06.003 15519506

[34] Ghiasvand R Rueegg CS Weiderpass E Green AC Lund E Veierød MB. Indoor Tanning and Melanoma Risk: Long-Term Evidence From a Prospective Population-Based Cohort Study. Am J Epidemiol (2017) 185(3):147–56. 10.1093/aje/kww148 28077359

[35] Hungarian National Cancer Registry. . https://onkol.hu/nemzeti-rakregiszter.

[36] Stratigos AJ Forsea AM van der Leest RJT de Vries E Nagore E Bulliard J-L. Euromelanoma: a dermatology-led European campaign against nonmelanoma skin cancer and cutaneous melanoma. Past Present Future Br J Dermatol (2012) 167(Suppl;2):99–104. 10.1111/j.1365-2133.2012.11092.x 22881594

[37] van der Leest RJ de Vries E Bulliard JL Paoli J Peris K Stratigos AJ. The Euromelanoma skin cancer prevention campaign in Europe: characteristics and results of 2009 and 2010. J Eur Acad Dermatol Venereol (2011) 25(12):1455–65. 10.1111/j.1468-3083.2011.04228.x 21951235

[38] Suppa M Gandini S Njimi H Bulliard JL Correia O Duarte AF. Association of sunbed use with skin cancer risk factors in Europe: an investigation within the Euromelanoma skin cancer prevention campaign. J Eur Acad Dermatol Venereol (2019) 33 Suppl 2:76–88. 10.1111/jdv.15307 30811689

[39] Ócsai H Ócsai H Battyáni Z Emri G Liszkay G Somlai B. EuroMelanoma Nappal szerzett tapasztalataink az elmúlt 5 évben (2009-2013). Magyar Onkológia (2013) 57(5):68. 23795350

[40] Petrovszky I Csányi I Szűcs M Ócsai H Houshmand N Kemény L. Factors influencing early detection of malignant melanoma. Orv Hetil (2016) 157(51):2028–33. 10.1556/650.2016.30610 27989229

[41] Balatoni T Liszkay G Miklós Z Kásler M. A melanoma malignum epidemiológiája (Klinikai tapasztalatok az Országos Onkológiai Intézetben) [Epidemiology of malignant melanoma (Clinical experience at the National Institute of Oncology in Hungary)]. Orv Hetil (2011) 152(25):1000–6. 10.1556/OH.2011.29148 21642052

[42] Janka EA Kékedi K Várvölgyi T Gellén E Kiss B Remenyik É. Increasing melanoma incidence in the elderly in North-East Hungary: is this a more serious problem than we thought? Eur J Cancer Prev (2019) 28(6):544–50. 10.1097/CEJ.0000000000000489 30399042

[43] OMSZ. Change in annual amounts of biologically effective UV radiation between 1995 and 2019. Hungarian Meteorology Institute (2020). Available at: https://www.met.hu/idojaras/humanmeteorologia/uv-b/uv_valtozas/.

[44] Paulson KG Gupta D Kim TS Veatch JR Byrd DR Bhatia S. Age-Specific Incidence of Melanoma in the United States. JAMA Dermatol (2019) 156(1):57–64. 10.1001/jamadermatol.2019.3353 PMC6865303 31721989

[45] Iannacone MR Green AC. Towards skin cancer prevention and early detection: evolution of skin cancer awareness campaigns in Australia. Melanoma Manag (2014) 1(1):75–84. 10.2217/mmt.14.6 30190812 PMC6094686

[46] Youl PH Soyer HP Baade PD Marshall AL Finch L Janda M. Can skin cancer prevention and early detection be improved via mobile phone text messaging? A randomised, attention control trial. Prev Med (2015) 71:50–6. 10.1016/j.ypmed.2014.12.009 25524612

[47] del Marmol V de Vries E van der Endt J Roseeuw D Vandaele M Pirard C. Evaluation of 7 years of Euromelanoma day, a voluntary melanoma screening campaign in Belgium. Melanoma Res (2006) 16:S22–3. 10.1097/00008390-200609001-00037

[48] Paoli J Danielsson M Wennberg AM. Results of the ‘Euromelanoma Day’ screening campaign in Sweden 2008. J Eur Acad Dermatol Venereol (2009) 23(11):1304–10. 10.1111/j.1468-3083.2009.03316.x 19522711

[49] Suppa M Altomare G Cannavò SP Capizzi R Catricalà C Colombo E. The Italian Euromelanoma Day: evaluation of results and implications for future prevention campaigns. Int J Dermatol (2014) 53(6):699–706. 10.1111/j.1365-4632.2012.05783.x 23230843

[50] Køster B Thorgaard C Philip A Clemmensen H. Sunbed use and campaign initiatives in the Danish population, 2007-2009: a cross-sectional study. J Eur Acad Dermatol Venereol (2011) 25(11):1351–5. 10.1111/j.1468-3083.2010.03960.x 21711466

[51] Diehl K Görig T Greinert R Breitbart EW Schneider S. Trends in Tanning Bed Use, Motivation, and Risk Awareness in Germany: Findings from Four Waves of the National Cancer Aid Monitoring (NCAM). Int J Environ Res Public Health (2019) 16(20):3913. 10.3390/ijerph16203913 PMC6843619 31618885

[52] Robert C Long GV Brady B Dutriaux C Maio M Mortier L. Nivolumab in previously untreated melanoma without BRAF mutation. N Engl J Med (2015) 372(4):320–30. 10.1097/01.cmr.0000136710.75287.1c 25399552

[53] Robert C Schachter J Long GV Arance A Grob J-J Mortier L. Pembrolizumab versus Ipilimumab in Advanced Melanoma. N Engl J Med (2015) 372(26):2521–32. 10.1056/NEJMoa1503093 25891173

[54] Donia M Ellebaek E Øllegaard TH Duval L Aaby JB Hoejberg L. The real-world impact of modern treatments on the survival of patients with metastatic melanoma. Eur J Cancer (2019) 108:25–32. 10.1016/j.ejca.2018.12.002 30605822

[55] Bosetti C La Vecchia C Naldi L Lucchini F Negri E Levi F. Mortality from cutaneous malignant melanoma in Europe. Has the epidemic levelled off? Melanoma Res (2004) 14(4):301–9. 10.1097/01.cmr.0000136710.75287.1c 15305162

