# Supplementary figures and images for: Changing Trends in Melanoma Incidence and Decreasing Melanoma Mortality in Hungary Between 2011 and 2019: A Nationwide Epidemiological Study
Source: Front Oncol. 2021 Feb 12;10:612459. doi: 10.3389/fonc.2020.612459 (PMC7908827; doi:10.3389/fonc.2020.612459)

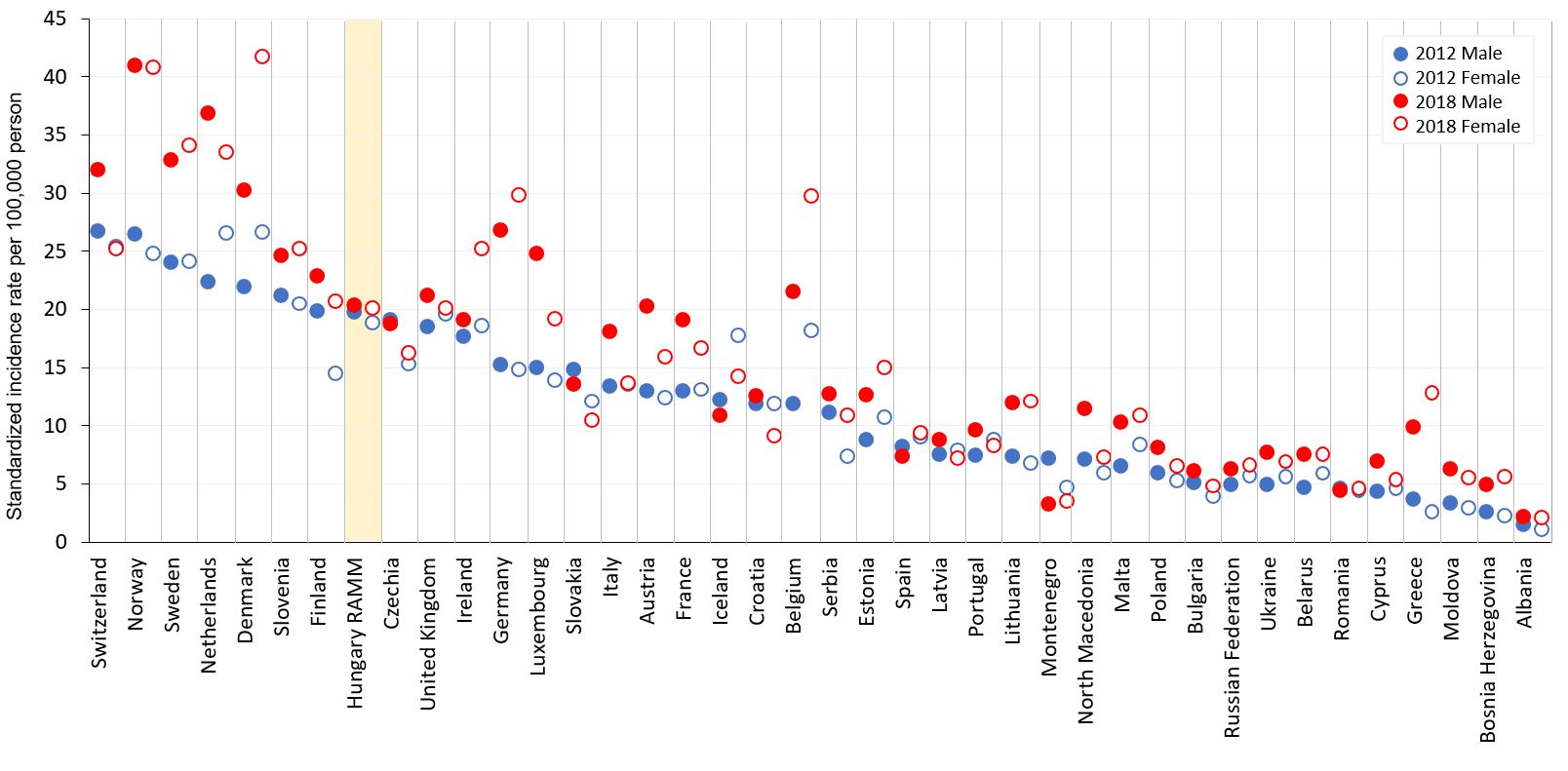

Supplement: Supplementary Figure 1 — Melanoma incidence rate of European countries in 2012 and 2018 by sex based on Ferlay reports (6, 7). [file Image_1.jpeg]
