# Supplementary material for: Changing Trends in Melanoma Incidence and Decreasing Melanoma Mortality in Hungary Between 2011 and 2019: A Nationwide Epidemiological Study
Source: Front Oncol. 2021 Feb 12;10:612459. doi: 10.3389/fonc.2020.612459 (PMC7908827; doi:10.3389/fonc.2020.612459)
Supplement: Supplementary file 2 [file DataSheet_1.docx]

**Supplementary Table 1** Mean age of melanoma patients at diagnosis and at the time of death (all-cause mortality). MM: malignant melanoma.

|  | **2011** | | **2012** | | **2013** | | **2014** | | **2015** | | **2016** | | **2017** | | **2018** | | **2019** | |
| --- | --- | --- | --- | --- | --- | --- | --- | --- | --- | --- | --- | --- | --- | --- | --- | --- | --- | --- |
| **Mean age at diagnosis (y, mean ±SD)** |  |  |  |  |  |  |  |  |  |  |  |  |  |  |  |  |  |  |
| Incident MM population | 60.81 | 16.05 | 61.09 | 16.29 | 60.84 | 16.24 | 60.40 | 16.70 | 60.55 | 16.55 | 60.32 | 16.59 | 60.88 | 16.19 | 60.81 | 16.72 | 61.11 | 16.10 |
| Male | 61.84 | 15.18 | 62.56 | 15.60 | 62.49 | 15.30 | 61.79 | 16.07 | 63.55 | 15.15 | 62.60 | 15.24 | 62.23 | 15.58 | 62.95 | 15.63 | 63.45 | 15.08 |
| Female | 59.80 | 16.80 | 59.85 | 16.76 | 59.31 | 16.93 | 59.13 | 17.16 | 57.78 | 17.29 | 58.25 | 17.48 | 59.58 | 16.67 | 58.92 | 17.39 | 58.91 | 16.71 |
| **Mean age of prevalent MM population (y, mean ±SD)** |  |  |  |  |  |  |  |  |  |  |  |  |  |  |  |  |  |  |
| Total MM population | 60.59 | 15.48 | 61.14 | 15.57 | 61.57 | 15.60 | 61.88 | 15.67 | 62.20 | 15.74 | 62.42 | 15.76 | 62.74 | 15.74 | 62.96 | 15.75 | 63.24 | 15.70 |
| Male | 62.14 | 14.92 | 62.63 | 15.04 | 63.07 | 15.08 | 63.32 | 15.17 | 63.86 | 15.19 | 64.10 | 15.16 | 64.31 | 15.18 | 64.54 | 15.16 | 64.83 | 15.08 |
| Female | 59.44 | 15.79 | 60.05 | 15.86 | 60.46 | 15.89 | 60.80 | 15.94 | 60.95 | 16.03 | 61.16 | 16.08 | 61.55 | 16.04 | 61.78 | 16.07 | 62.05 | 16.04 |
| **Mean age at death (y, mean ±SD)** |  |  |  |  |  |  |  |  |  |  |  |  |  |  |  |  |  |  |
| Total MM population | 69.77 | 13.14 | 70.80 | 13.74 | 72.71 | 13.54 | 71.82 | 13.70 | 74.15 | 13.29 | 73.80 | 13.44 | 75.35 | 12.56 | 75.22 | 12.97 | 76.37 | 12.26 |
| Male | 69.33 | 12.67 | 69.57 | 13.17 | 71.85 | 13.53 | 70.70 | 12.75 | 72.87 | 13.03 | 73.44 | 12.89 | 74.52 | 12.84 | 74.65 | 13.06 | 75.50 | 11.67 |
| Female | 70.42 | 13.82 | 72.47 | 14.32 | 73.79 | 13.48 | 73.10 | 14.62 | 76.01 | 13.45 | 74.27 | 14.13 | 76.39 | 12.13 | 75.93 | 12.83 | 77.50 | 12.91 |

**Supplementary Table 2** Age-standardized incidence rates of melanoma in Hungary by sex and between 2011 and 2019 using ESP 2013 for standardization. CI: confidence interval; ESP: European Standard Population.

| **Incidence rate per 100,000 person year at risk** | **2011** | **2012** | **2013** | **2014** | **2015** | **2016** | **2017** | **2018** | **2019** |
| --- | --- | --- | --- | --- | --- | --- | --- | --- | --- |
| Total melanoma population | 25.61 | 24.76 | 26.04 | 26.43 | 28.77 | 27.50 | 27.15 | 25.65 | 24.39 |
| Lower 95%CI | 24.59 | 23.76 | 25.02 | 25.40 | 27.70 | 26.45 | 26.13 | 24.65 | 23.41 |
| Upper 95%CI | 26.62 | 25.76 | 27.06 | 27.45 | 29.83 | 28.54 | 28.18 | 26.65 | 25.36 |
| Male melanoma population | 31.22 | 28.28 | 30.94 | 31.01 | 34.57 | 32.18 | 32.58 | 29.11 | 28.76 |
| Lower 95%CI | 29.45 | 26.59 | 29.19 | 29.27 | 32.73 | 30.42 | 30.82 | 27.45 | 27.11 |
| Upper 95%CI | 32.99 | 29.97 | 32.69 | 32.75 | 36.42 | 33.94 | 34.34 | 30.77 | 30.41 |
| Female melanoma population | 22.63 | 23.61 | 23.91 | 24.48 | 26.72 | 25.81 | 24.74 | 24.60 | 22.72 |
| Lower 95%CI | 21.36 | 22.32 | 22.61 | 23.17 | 25.35 | 24.46 | 23.42 | 23.29 | 21.45 |
| Upper 95%CI | 23.90 | 24.90 | 25.21 | 25.79 | 28.09 | 27.16 | 26.06 | 25.92 | 23.98 |

**Supplementary Table 3** Mean annual and total changes in age-standardized melanoma incidence rates in different study periods (2011–2019; 2011–2015 and 2015–2019), and change of trends of melanoma incidence rates between 2015–2019 and 2011–2015. CI: confidence interval.

| **Mean annual change % (95% CI) p-value** | | | | | | |
| --- | --- | --- | --- | --- | --- | --- |
|  | 2011-2019 | | 2011-2015 | | 2015-2019 | |
| Overall | 0.11 (-2.74 - 2.93) | 0.976 | 3.76 (1.28 - 8.87) | 0.009 | -3.00 (-4.18 - -0.63) | 0.035 |
| Female | 0.49 (-2.22 - 3.36) | 0.495 | 4.40 (2.53 - 9.27) | <0.001 | -2.97 (-4.08 - -0.77) | 0.004 |
| Male | -0.09 (-3.21 - 3.30) | 0.782 | 3.81 (-0.29 - 11.13) | 0.067 | -3.36 (-5.50 - 0.92) | 0.062 |
| **Total change % (95% CI) p-value** | | | | | | |
|  | 2011-2019 | | 2011-2015 | | 2015-2019 | |
| Overall | 0.84 (-19.92 - 25.97) | 0.976 | 15.92 (5.24 - 40.47) | 0.009 | -11.45 (-15.70 - -2.50) | 0.035 |
| Female | 3.97 (-16.44 - 30.23) | 0.495 | 18.82 (10.5 - 42.58) | <0.001 | -11.35 (-15.35 - -3.05) | 0.004 |
| Male | -0.70 (-23.00 - 29.61) | 0.782 | 16.14 (-1.17 - 52.53) | 0.067 | -12.77 (-20.25 - 3.73) | 0.062 |
| **Trend change % (95% CI) p-value** | | | | | | |
|  | 2015-2019 vs 2011-2015 | |  | |  | |
| Overall | -6.51 (-11.65 - -2.25) | 0.011 |  |  |  |  |
| Female | -7.06 (-11.77 - -3.54) | 0.002 |  |  |  |  |
| Male | -6.91 (-14.23 - 0.69) | 0.063 |  |  |  |  |

**Supplementary Table 4** Age-standardized mortality rates of melanoma in Hungary by sex between 2011 and 2018 using ESP 2013 for standardization. CI: confidence interval; ESP: European Standard Population.

| Mortality rate per 100,000 person year at risk | **2011** | **2012** | **2013** | **2014** | **2015** | **2016** | **2017** | **2018** | **2019** |
| --- | --- | --- | --- | --- | --- | --- | --- | --- | --- |
| Total melanoma population | 3.91 | 4.10 | 3.77 | 4.04 | 3.73 | 3.57 | 3.53 | 3.30 |  |
| Lower 95%CI | 3.51 | 3.69 | 3.38 | 3.64 | 3.34 | 3.19 | 3.15 | 2.94 |  |
| Upper 95%CI | 4.32 | 4.52 | 4.17 | 4.45 | 4.12 | 3.96 | 3.91 | 3.67 |  |
| Male melanoma population | 5.85 | 5.69 | 5.09 | 5.28 | 5.98 | 5.57 | 5.09 | 4.73 |  |
| Lower 95%CI | 5.06 | 4.92 | 4.35 | 4.54 | 5.18 | 4.79 | 4.36 | 4.03 |  |
| Upper 95%CI | 6.63 | 6.46 | 5.82 | 6.03 | 6.78 | 6.34 | 5.81 | 5.42 |  |
| Female melanoma population | 2.56 | 2.97 | 2.83 | 3.20 | 2.33 | 2.37 | 2.39 | 2.40 |  |
| Lower 95%CI | 2.15 | 2.52 | 2.39 | 2.73 | 1.94 | 1.98 | 2.01 | 2.00 |  |
| Upper 95%CI | 2.97 | 3.42 | 3.26 | 3.66 | 2.73 | 2.77 | 2.78 | 2.80 |  |

**Supplementary Table 5** Mean annual and total changes in age-standardized melanoma mortality rates in the different study periods (2011­–2018; 2011–2015 and 2015–2018), and change of trends in melanoma mortality rates between the 2015–2018 and 2011–2015. CI: confidence interval.

| **Mean annual change % (95% CI) p-value** | | | | | | |
| --- | --- | --- | --- | --- | --- | --- |
|  | 2011-2018 | | 2011-2015 | | 2015-2018 | |
| Overall | -2.55 (-4.41 - -0.67) | 0.013 | -1.39 (-7.71 - 1.73) | 0.208 | -4.69 (-7.46 - -2.35) | 0.007 |
| Female | -2.98 (-7.95 - 1.2) | 0.094 | -3.22 (-26.8 - 6.87) | 0.532 | -4.95 (-14.47 - 1.04) | 0.302 |
| Male | -1.79 (-5.24 - 2.34) | 0.233 | 1.24 (-4.72 - 13.41) | 0.747 | -4.77 (-7.7 - 1.78) | 0.143 |
| **Total change % (95% CI) p-value** | | | | | | |
|  | 2011-2018 | | 2011-2015 | | 2015-2018 | |
| Overall | -16.55 (-27.07 - -4.59) | 0.013 | -5.45 (-27.46 - 7.12) | 0.208 | -13.43 (-20.76 - -6.87) | 0.007 |
| Female | -19.1 (-44 - 8.69) | 0.094 | -12.28 (-71.28 - 30.44) | 0.532 | -14.13 (-37.44 - 3.15) | 0.302 |
| Male | -11.86 (-31.4 - 17.59) | 0.233 | 5.07 (-17.57 - 65.45) | 0.747 | -13.65 (-21.37 - 5.44) | 0.143 |
| **Trend change % (95% CI) p-value** | | | | | | |
|  | 2015-2018 vs 2011-2015 | |  | |  | |
| Overall | -3.35 (-9.20 - 4.59) | 0.163 |  |  |  |  |
| Female | -1.79 (-17.24 - 38.03) | 0.779 |  |  |  |  |
| Male | -5.94 (-18.52 - 5.49) | 0.312 |  |  |  |  |

**Supplementary Table 6 (for Figure 4)** Age-standardized incidence and mortality rates of European countries from 2018 based on Ferlay’s publication (using ESP 1976). ESP: European Standard Population.

| **ESP 1976 standard population  Country** | **Incidence rate per 100,000 person year at risk** | | **Mortality rate per 100,000 person year at risk** | |
| --- | --- | --- | --- | --- |
|  | **Male** | **Female** | **Male** | **Female** |
| Europe | 15.8 | 14.6 | 3.2 | 1.9 |
| EU-28 | 18.4 | 17.7 | 3.2 | 1.9 |
| Central Eastern Europe | 7.6 | 7.1 | 3.0 | 2.0 |
| Belarus | 7.6 | 7.5 | 3.0 | 1.8 |
| Bulgaria | 6.2 | 4.8 | 2.6 | 1.5 |
| Czech Republic | 18.8 | 16.2 | 3.4 | 1.8 |
| Hungary | 14.0 | 13.2 | 2.9 | 2.1 |
| Hungary NHIF | 20.3 | 20.0 | 3.0 | 1.7 |
| Moldova | 6.3 | 5.5 | 2.8 | 1.7 |
| Poland | 8.2 | 6.5 | 4.0 | 2.4 |
| Romania | 4.5 | 4.6 | 1.9 | 1.4 |
| Russian Fed | 6.3 | 6.6 | 2.7 | 2.0 |
| Slovakia | 13.6 | 10.5 | 4.8 | 3.2 |
| Ukraine | 7.7 | 6.9 | 3.4 | 1.9 |
| Northern Europe | 23.4 | 23.2 | 3.8 | 2.2 |
| Denmark | 30.3 | 41.7 | 4.1 | 3.1 |
| Estonia | 12.7 | 15.0 | 3.7 | 2.1 |
| Finland | 22.9 | 20.7 | 4.4 | 1.7 |
| Iceland | 11.0 | 14.3 | 2.2 | 2.0 |
| Ireland | 19.2 | 25.2 | 3.4 | 2.4 |
| Latvia | 8.9 | 7.2 | 3.0 | 2.2 |
| Lithuania | 12.1 | 12.1 | 2.9 | 1.8 |
| Norway | 41.1 | 40.8 | 6.3 | 4.1 |
| Sweden | 32.9 | 34.1 | 4.8 | 2.6 |
| United Kingdom | 21.2 | 20.1 | 3.5 | 1.9 |
| Southern Europe | 12.5 | 11.2 | 2.7 | 1.6 |
| Albania | 2.2 | 2.1 | 0.8 | 0.6 |
| Bosnia Herzegovina | 5.0 | 5.6 | 2.2 | 1.5 |
| Croatia | 12.6 | 9.2 | 4.6 | 2.5 |
| Cyprus | 7.0 | 5.4 | 3.0 | 1.4 |
| Greece | 10.0 | 12.8 | 2.3 | 1.6 |
| Italy | 18.1 | 13.7 | 3.1 | 1.6 |
| Macedonia | 11.5 | 7.3 | 4.0 | 2.2 |
| Malta | 10.4 | 10.9 | 2.2 | 1.4 |
| Montenegro | 3.3 | 3.5 | 1.2 | 1.3 |
| Portugal | 9.7 | 8.3 | 2.5 | 1.6 |
| Serbia | 12.8 | 10.9 | 4.7 | 2.6 |
| Slovenia | 24.7 | 25.2 | 4.8 | 3.2 |
| Spain | 7.4 | 9.4 | 1.9 | 1.3 |
| Western Europe | 24.9 | 25.0 | 3.3 | 1.9 |
| Austria | 20.4 | 15.9 | 3.4 | 1.8 |
| Belgium | 21.6 | 29.7 | 2.9 | 2.6 |
| France | 19.1 | 16.7 | 3.0 | 1.7 |
| Germany | 26.9 | 29.9 | 3.2 | 1.8 |
| Luxembourg | 24.9 | 19.2 | 3.1 | 1.7 |
| Netherlands | 37.0 | 33.5 | 4.6 | 3.1 |
| Switzerland | 32.1 | 25.2 | 4.2 | 1.8 |
